# Genetic Structure and Diversity of the Endangered Fir Tree of Lebanon (*Abies cilicica* Carr.): Implications for Conservation

**DOI:** 10.1371/journal.pone.0090086

**Published:** 2014-02-27

**Authors:** Lara Awad, Bruno Fady, Carla Khater, Anne Roig, Rachid Cheddadi

**Affiliations:** 1 University of Montpellier II, Institute of Evolutionary Sciences, CNRS UMR 5554, Montpellier, France; 2 INRA, UR629, Ecologie des Forêts Méditerranéennes (URFM), Domaine St Paul, Avignon, France; 3 Center for Remote Sensing, National Council for Scientific Research-Lebanon, Bir Hassan, Beirut, Lebanon; CNR, Italy

## Abstract

The threatened conifer *Abies cilicica* currently persists in Lebanon in geographically isolated forest patches. The impact of demographic and evolutionary processes on population genetic diversity and structure were assessed using 10 nuclear microsatellite loci. All remnant 15 local populations revealed a low genetic variation but a high recent effective population size. *F_ST_*-based measures of population genetic differentiation revealed a low spatial genetic structure, but Bayesian analysis of population structure identified a significant Northeast-Southwest population structure. Populations showed significant but weak isolation-by-distance, indicating non-equilibrium conditions between dispersal and genetic drift. Bayesian assignment tests detected an asymmetric Northeast-Southwest migration involving some long-distance dispersal events. We suggest that the persistence and Northeast-Southwest geographic structure of *Abies cilicica* in Lebanon is the result of at least two demographic processes during its recent evolutionary history: (1) recent migration to currently marginal populations and (2) local persistence through altitudinal shifts along a mountainous topography. These results might help us better understand the mechanisms involved in the species response to expected climate change.

## Introduction

Geographic patterns of genetic variation in conifers have long been studied at the species and populations levels [1]–[9]. Geographic range size, geographic isolation and biogeographic position may play a crucial role in shaping patterns of genetic variation [10]. Widely distributed woody plant species with high longevity and high fecundity generally retain high genetic diversity within populations but low genetic differentiation among populations [11], [12]. The opposite is generally found for endemic and narrowly distributed woody plants with the same combination of traits [12]. However, the low genetic variation found in rare species may be an overgeneralization [13]. For instance, high gene diversity and population structure can be found in endemic species with restricted geographic range [14]. The imprint of geographic range size on patterns of genetic diversity may also be indirectly driven by species' dispersal ability. At the end of the Pleistocene, the rapid geographical range expansion of many tree species was characterized by the occurrence of long-distance dispersal events [15]. Consequences of such range expansions on spatial genetic patterns and on the maintenance or erosion of genetic diversity may respectively depend on the forms of dispersal [16] and on the number of long-distance dispersal events [17]. Gene flow arising from dispersal can either impede or promote evolution depending on the geographic structure of natural populations [18]. For instance, local adaptation may be achieved despite high gene flow and long-distance dispersal in expanding populations [19], [20].

The genetic imprint of geographic isolation, or fragmentation, on natural populations can vary with respect to their demographic structure. Relict populations in fragmented landscapes predictably show low genetic variation but high genetic differentiation due to spatial isolation [21]. Demographic bottlenecks resulting from fragmentation may not be accompanied by genetic bottlenecks due to temporal fluctuation in population sizes, mating system dynamics, or metapopulation structure [22], [23], [24]. Short-range dispersal occurring between neighboring populations should contribute to the genetic differentiation of remote populations via isolation-by-distance [25]. Over time, the isolation-by-distance genetic pattern should be pronounced at equilibrium between dispersal and genetic drift in long-established populations [26]. In demographically unstable populations, however, fragmentation can either increase or decrease the genetic variance among populations. The outcome may depend on the mode of colonization [27], on temporal and spatial variation and co-variation in demographic parameters [28], or on the rates of extinction and recolonization relative to the effective population size [18] or relative to the effective number of migrants [27], [29].

The biogeographic position within a geographic range or a biome may impact genetic diversity within and among populations. In the Mediterranean basin, genetic diversity is higher than other regions of the world for conifers [30] and the rear-edge populations of temperate tree species often display large regional genetic diversity and unique genetic variants [31]. Most rear-edge populations have not been involved in major postglacial recolonization events [32]. Instead, they have persisted locally with small altitudinal shifts in range [33], [34]. Longitude and underlying demographic processes such as climate-related effective population size changes and migration can also explain genetic diversity gradients. For instance, a pattern of decreasing within-population genetic diversity is observable from east to west across the Mediterranean basin in a wide range of taxa [10], [35].

**Figure 1:**
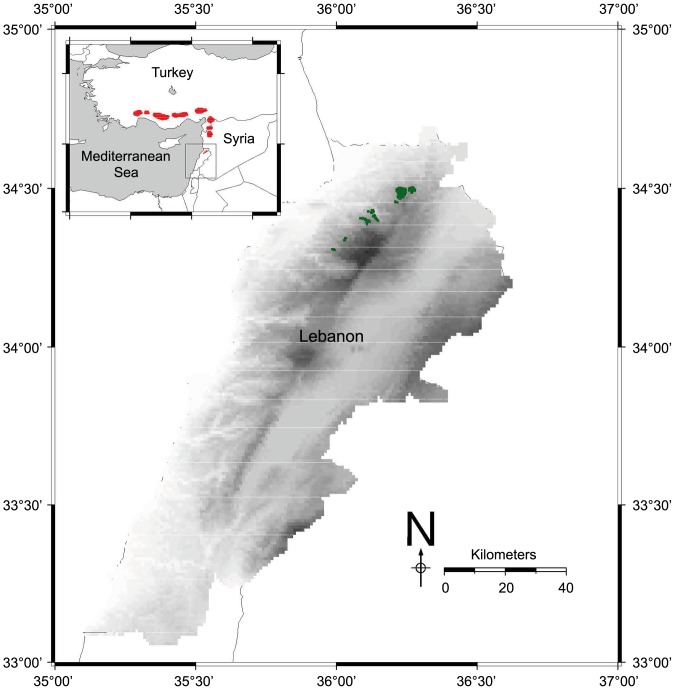
Range of distribution of *Abies cilicica* in the Eastern Mediterranean. Remnant populations of *Abies cilicica* in Turkey, Syria and Lebanon are plotted in red onto the map of the Eastern Mediterranean. Remnant populations of *Abies cilicica* in Lebanon are plotted in green onto the map of Lebanon where they are currently found on the western slopes of the northern part of Mount Lebanon. Geographical position of *Abies cilicica* in Turkey and Syria were plotted from a euforgen map [105] while the geographical position of *Abies cilicica* in Lebanon corresponds to our sampling data. Map was prepared using qgis version 1.8.0 [106] and the gmt package [107] within r [108].

In this study, we have explored the imprint of geographic range size, geographic isolation and biogeographic position and that of demographic processes such as dispersal ability and viability on the modern genetic diversity of *Abies cilicica* Carr. in Lebanon (Cilician fir). *Abies cilicica* is a long-lived and outcrossed conifer species. It is endemic to the Eastern Mediterranean in the Taurus, Anti-Taurus and Amanus mountains of Turkey, the Alawi mountains in Syria and the Northern Mount Lebanon range in Lebanon (Figure 1). However, its range is not continuous, and in Lebanon, noticeably, it is geographically restricted and geographically isolated from the rest of its range with more than a 150 km distance from *Abies cilicica* in Syria (Figure 1). In Lebanon, *Abies cilicica* is geographically marginal as it currently occupies the southernmost geographical position of its range (Figure 1). The varied topography of Mount Lebanon shelters geographically isolated forest patches. Historically, Ancient Egyptian texts attest the use of *Abies cilicica* for its timber, during the Ancient Egyptian New Kingdom, as a sign of power of the Pharaoh, particularly in the construction of flagpoles for temple pylons, in shipbuilding, and in the construction of the Barque of Amun [36]. During World War II, the wood of *Abies cilicica* was cut mainly from Qammouaa to build the railroad between Tripoli and Haifa [37]. Major current anthropogenic disturbances include illegal logging and overgrazing activities. The IUCN status of *Abies cilicica* is Near Threatened overall [38], but IUCN considers it critically endangered at national level in Lebanon.

Because genetic diversity is potentially high in the Eastern Mediterranean and for mountain species [10], and because its distribution range is patchy and some populations display low census size, *Abies cilicica* in Lebanon offers the opportunity to answer the following questions for which accumulating experimental evidence remains central: (1) Is biogeographic position affecting genetic diversity within and among populations?, (2) Is habitat fragmentation increasing spatial genetic structure?, (3) Is geographic isolation limiting dispersal?, and (4) Is geographic marginality affecting persistence?

## Material and Methods

### Ethics Statement

All the field sampling of *Abies cilicica* in Lebanon was carried out after obtaining mission permits from CNRS in France and NCSR in Lebanon (number L03504, L03701, 2938/w and 5170/w) and following the approval in Lebanon of corresponding municipalities and nature reserves organizational teams. Sampling of *Abies marocana* Trab. (also known as *Abies pinsapo* subsp. *marocana* Trab.) was permitted by the Talassemtane National Park, Morocco. Needle samples of *Abies bornmuelleriana* Mattf., *Abies cephalonica* Loud. and *Abies alba* Mill. were provided by INRA-URFM.

### Sampling Area and Plant Material

*Abies cilicica* is currently distributed in Lebanon across 15 remnant populations located between ca. 1000–1800 m.a.s.l. on the western slopes of northern Mount Lebanon. Moist airstreams off the Mediterranean approaching over Mount Lebanon result in a classic orographic precipitation pattern. Rain falling on the western side of Mount Lebanon becomes snow at higher elevations during winter. The species currently occupies humid bioclimatic zones with a mean annual precipitation over 800 mm. The varied topography of Mount Lebanon allows it to inhabit both the Supra-Mediterranean (1000–1500 m.a.s.l.) and Mountain-Mediterranean (1500–2000 m.a.s.l.) vegetation zones. Currently, it occupies a patchy distribution covering 1613 ha equivalent to 1.2% of the total forest cover of Lebanon, compared to 1.58% for *Cedrus libani* A. Rich. (Cedar of Lebanon) [39].

We have exhaustively sampled all the 15 remnant natural populations of *A. cilicica* in Lebanon (Table 1, Table S1). Adult trees were sampled randomly at distance intervals of 20 m approximately keeping sampled areas to a minimum. A total of 351 leaf samples of *A. cilicica* from Lebanon were collected and then conserved at −20°C prior to DNA extraction.

**Table 1:** Origin, location and sample size of *Abies cilicica* populations in Lebanon.

Code	Location	Longitude	Latitude	Altitude*	Sample size
KCHB^1^	Kobayat	36° 15.720′ E	34° 29.881′ N	1582–1758	30
BTJF^1^	Hermel	36° 16.643′ E	34° 29.731′ N	1538–1593	30
CHIR^1^	Qammouaa	36° 14.533′ E	34° 29.790′ N	1553–1635	28
JBMD^1^	Qammouaa	36° 13.594′ E	34° 29.099′ N	1394–1581	30
MKTF^1^	Qammouaa	36° 13.580′ E	34° 28.101′ N	1548–1597	30
NBSH^1^	Qammouaa	36° 12.626′ E	34° 27.404′ N	1460–1545	30
QARS^2^	Qarsita	36° 06.849′ E	34° 25.670′ N	1037–1140	11
QJRN^1^	Jaïroun	36° 07.610′ E	34° 25.549′ N	1304–1439	28
QEMN^1^	Wadi Jhanam	36° 08.056′ E	34° 24.846′ N	1102–1191	28
HNKT^1^	Kfarbnine	36° 05.010′ E	34° 24.353′ N	1389–1453	12
HNKF^1^	Kfarbnine	36° 06.300′ E	34° 24.081′ N	1422–1505	21
GHAB^1^	Kfarbnine	36° 06.484′ E	34° 23.953′ N	1480–1597	24
HKYT^2^	Bqaa Safrine	36° 01.886′ E	34° 20.649′ N	1551–1695	12
KMHR^2^	Karm El Mohr	36° 01.585′ E	34° 20.186′ N	1639–1704	18
EHDN^2^	Ehden	35° 59.533′ E	34° 18.460′ N	1447–1613	19

*Elevation in meters above sea level.

1 membership of the local population to deme 1 (Northeastern ridge).

2 membership of the local population to deme 2 (Southwestern ridge).

### DNA Extraction

Total genomic DNA was successfully extracted and purified from 45 mg of needles per sample using the QIAGEN DNeasy 96 plant kit following manufacturer's protocol. Furthermore, 48 needle samples from four Mediterranean *Abies* species were also used for DNA extraction to serve as reference for the genetic analyses. These included 6 samples of *A. marocana* from the Talassemtane National Park in Morocco, 8 samples representing a mixture of 6 populations of *A. bornmuelleriana* from Turkey, 8 samples representing a mixture of 8 populations of *A. cephalonica* from Greece, 10 samples representing a mixture of 10 populations of *A. alba* from the French Pyrenees and 16 samples representing a mixture of 4 populations of *A. alba* from the French Southern Alps (Table S1).

### Microsatellite Analysis

Microsatellites are efficient molecular markers for describing the demographic history of populations [40], and therefore addressing specific questions related to their evolutionary ecology and conservation [41]. Genomic DNA amplification was performed using 10 nuclear microsatellites: NFF3, NFH15, NFH3 and NFF7 developed for *A. nordmanniana* Stev. [42], and SF1, SFb4, SFb5, SF50, SF78 and SF333 developed for *A. alba* Mill. [43]. The forward primer was 5′ end labeled by one of fluorescent dyes 6-FAM (blue), HEX (green), TAMRA (yellow) or ATTO565 (red) (Eurofins MWG Operon). Two multiplex microsatellite sets (NFF3, NFH15, NFH3, NFF7 and SF1, SFb4, SFb5, SF50, SF78, SF333) for which allele drop-out was controlled, were amplified for all individuals by polymerase chain reaction (PCR) using Qiagen Multiplex PCR Kit. Multiplex PCR was run in an 11 µL reaction on a Mastercycler EP Gradient S96 thermal cycler (Eppendorf). The PCR mix contained 2× Qiagen Multiplex PCR Master Mix (1× final concentration), 0.2 µM of each forward and reverse primers, 5× Q-Solution (0.5× final concentration), RNase-free water (Qiagen) qs to 9 µL and 2 µL (about 20 ng) of genomic DNA. The thermal cycling consisted of initial denaturation step at 95°C for 15 minutes; 3-step cycling repeated 30 times and consisting of denaturation at 94°C for 30 seconds, annealing at 57°C for 90 seconds and extension at 72°C for 60 seconds; and a final extension step at 72°C for 10 minutes.

Capillary electrophoresis of multiplexed PCR products was performed on an ABI 3730XL automated sequencer (Applied Biosystems) at INRA Avignon, with the GeneScan™ - 500 LIZ™ size standard (Applied Biosystems) run with each sample. In order to decrease the risks of genotyping errors related to automated or arbitrary decisions in allelic binning [44], [45], allelic binning and scoring of genotypes were performed manually by two different persons using the software GeneMapper 4.1 (Applied Biosystems) and compared to generate the final data set. The genotype dataset for the 10 microsatellites of 399 individuals of *A. cilicica*, *A. cephalonica*, *A. bornmuelleriana*, *A. marocana* and *A. alba* is publicly deposited in the DRYAD repository.

## Data Analysis

### Hardy-Weinberg Equilibrium, Linkage Disequilibrium and Null Alleles

Exact tests for deviations from Hardy–Weinberg equilibrium (HWE) were performed using the heterozygote deficiency and heterozygote excess tests implemented in genepop 4.1.3 [46], [47]. Testing for linkage disequilibrium (LD) was also performed in genepop 4.1.3 by using the log likelihood ratio statistic test (*G*-test). Exact P-values for the individual population or locus tests were estimated using the Markov Chain algorithm implemented in genepop 4.1.3 with 10000 dememorization steps, 100 batches and 5000 iterations and were then corrected both using the sequential Bonferroni procedure [48] and the Benjamini-Hochberg method [49] to ensure controlling both the Family Wise Error Rate (FWER) and the False Discovery Rate (FDR). The presence and frequency of null alleles were examined using freena [50] following the Expectation Maximization (EM) algorithm [51] which was found to provide unbiased and low variance estimates of null allele frequencies [50]. Given that the presence of null alleles result in an overestimation of population genetic differentiation [50], freena was used to compute the *F_ST_* statistic [52] both using and without using the *ENA* (Excluding Null Alleles) correction method, i.e. by excluding or not excluding null allelic states from the *F_ST_* computation [50]. The bootstrap 95% confidence intervals (CI) for the global *F_ST_* values were calculated using 50000 replicates over loci.

### Within-Population Genetic Diversity

genalex v6 [53] and fstat version 2.9.3.2 [54] were used to estimate observed heterozygosity *H_O_*, expected heterozygosity *H_E_* [55], [56], rarefied allelic richness *A_R_* [57], [58] for a sample size of n = 20 gene copies (10 diploid trees), and inbreeding coefficient *F_IS_* [59]. The bootstrap 99% CI for the global *F_IS_* value over loci was calculated in fstat version 2.9.3.2 using 15000 replicates. Rarefied private allelic richness *П_s_* was computed using the rarefaction method [57], [60] implemented in hp-rare 1.1 [61] with a minimum sample size of n  =  20 gene copies. Statistical significance at the 5% nominal level of the difference between the mean value of *H_O_* and *H_E_* across all samples for all loci was verified using a Student's *t*-test. Differences in the within-population genetic diversity and allelic richness among *A. cilicica*, *A. marocana*, *A. bornmuelleriana*, *A. cephalonica* and *A. alba* were assessed using a permutation test implemented in fstat version 2.9.3.2 [54]. Statistical significance at the 5% nominal level was calculated after 10000 permutations of populations among groups.

### Among-Population Genetic Differentiation

The differentiation between populations was computed in spagedi [62] based on allele identity and allele size by respectively using *F_ST_* [59] and *R_ST_* [63], [64], and their significance was determined by 10000 permutations. The presence of a phylogeographic pattern was also assessed, using the same software, after 10000 randomizations of allele sizes among alleles within loci [65], by testing whether *R_ST_* computed before randomization was larger than *R_ST_* obtained after allele size permutation (*pR_ST_*). The randomization test also infers whether or not strict stepwise-like mutations contributed to population differentiation [65].

Population genetic structure was also assessed using two Bayesian clustering approaches with different underlying assumptions concerning the units of clustering (individual, population), the types of clustering (non-spatial, spatial), and the computation algorithm (Markov chain Monte Carlo (MCMC), stochastic optimization). The first Bayesian clustering approach, implemented in the software structure 2.3.3 [66], identifies the number of distinct genetic clusters (*K*) and probabilistically assigns individual multilocus genotypes to these clusters. To infer posterior probabilities of *K*, we ran a series of independent runs with different user-defined values of *K* clusters ranging from 1 to 15. Assuming that individuals may have mixed ancestry and that the different populations have correlated allele frequency [67], analysis was run ten times for 10^6^ iterations of MCMC after a burn-in period of 30000. No prior information on the sampling locations of each individual was used as recommended in the software documentation [68]. structure results were then processed in a web-based program, structure harvester [69], which implements the Evanno method [70] to estimate a mode at the true *K*. The second Bayesian approach, implemented in the software baps 6 [71], estimates the hidden population substructure by clustering sampled populations into panmictic groups [72]. Analysis was performed at the population level by using a spatial mixture model with prior information on the geographical position of the sampled populations, to prevent both over-smoothing in the case of a weak spatial structure and the emergence of spurious genetic structure as a result of weak stochastic fluctuations in the allele frequencies [73], [74]. Spatial genetic mixture analysis was run ten times after choosing a set of possible values of *K* clusters ranging from 1 to 15. To find the optimal partition, the program uses a stochastic optimization algorithm, which is less computationally intensive than the MCMC algorithm used in structure [75]. A joint posterior distribution of partitions of the sampled populations into genetically divergent groups was produced and then plotted onto the map of Lebanon.

fstat version 2.9.3.2 [54] was used to compute, for the identified clusters, the observed (*H_O_*) and expected (*H_E_*) proportion of heterozygotes and the overall gene diversity (*H_T_*) according to [76], *F_IS_* and *F_ST_* according to [59], and rarefied allelic richness (*A_R_*) for a sample size of n = 102 gene copies or 51 diploid individuals [57], [58]. Statistical significance at the 5% significance level was obtained after 10000 permutations of populations among demes. *R_ST_* [63], [64] was also computed among populations of each of the identified clusters using the software spagedi [62], as well as the occurrence of a phylogeographic pattern as described above.

Direct estimates of recent migration rates within and between the current clusters were generated in the software geneclass2 [77] through assignment tests allowing the detection of first-generation (F_0_) migrants. The likelihood (*L*) that an individual originates from a given population sample was computed using the statistical criterion 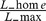, i.e. the ratio of the likelihood of finding a given individual in the population in which it was sampled to the greatest likelihood among all sampled populations [78]. Given that all the populations of *A. cilicica* in Lebanon have been extensively sampled, this criterion was considered to be informative on recent dispersal [77], [78]. The likelihood estimation was computed using the likelihood-based Bayesian method of [79], which has been shown to perform better than frequency-based and distance-based methods [80]. The probability computation, at the 1% significance level, was assessed by using the Monte Carlo resampling method of [78] with 10000 simulated individuals instead of the Monte Carlo resampling methods of [79], [80] to better control type I error rates.

### Isolation-By-Distance

Isolation-by-distance (IBD) [25] was analyzed using ibdws version 3.23 [81] by assessing the statistical significance, in a Mantel test [82] with 30000 permutations, of the relationship between the measure of similarity 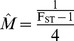 [26] among population pairs and the comparable matrix of geographic distances in a two-dimensional stepping stone model. This approach was chosen as it has been shown to distinguish between equilibrium and non-equilibrium populations under dispersal and genetic drift [26]. Both 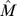 and the geographic distance were log-transformed as suggested in [26]. The strength of IBD was quantified using a reduced major axis (RMA) regression, which is more appropriate for this purpose than a standard ordinary least squares (OLS) regression [83].

### Genetic Bottlenecks

Three complementary methods were used to detect whether or not *A. cilicica* in Lebanon had experienced recent and severe genetic bottlenecks. The first method, implemented in the software m-p-val and critical_m [84], is based on the computation of the parameter 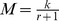, i.e. the mean ratio of the number of alleles (*k*) to the range in allele size (*r*), from a population sample of microsatellite loci [84], [85]. Along with the fact that when effective population size decreases, genetic drift intensifies and alleles are subsequently lost, this ratio is expected to be smaller in recently and severely bottlenecked populations than in populations under mutation-drift equilibrium [84]. This method can detect recent genetic bottlenecks over a period of time of 100 generations [84]. The analysis was run assuming a Two-Phase Mutation Model (TPM) [86] of microsatellite loci with parameters *p_s_* (proportion of one-step mutations) = 0.88 and *Δ_g_* (average size of non one-step mutations) = 2.8 as reviewed in [84]. In m-p-val, the average observed value of *M* calculated from the microsatellite dataset was compared with a simulated equilibrium distribution of *M* for different values of the population parameter 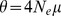 (where 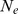 is the pre-bottleneck effective population size, and 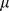 is the mutation rate of microsatellites per locus per generation). In critical_m, values of the critical *M* threshold (*M*_c_), below which a bottleneck is evident, were also calculated for the different *θ* values. Considering an average mutation rate per locus per generation equal to 0.0005 [84], [87], statistical significance was assessed with 10000 simulations carried out with values of *θ* Є {10; 1; 0.5; 0.1} corresponding respectively to a pre-bottleneck effective population size of 5000, 500, 250 and 50.

The second method makes use of a Wilcoxon's signed rank test to compare the distribution of the heterozygosity expected under mutation-drift equilibrium and under HWE and can detect recent genetic bottlenecks over the past 4 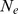 or 2 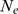 generations [88]. The third method is a mode-shift test, which searches for bottleneck-induced and transient distortions of allele frequency distributions over the past few dozen generations [89]. The last two methods were performed using the software bottleneck [90] and a Two-Phase Mutation Model (TPM) [86] consisting of 95% of Stepwise Mutation Model (SMM) [91] and 5% of Infinite Allele Model (IAM) [92] with a variance of 12% among multi-steps, as recommended in [90], and 10000 iterations.

## Results

### Hardy-Weinberg Equilibrium, Linkage Disequilibrium and Null Alleles

Out of a total of 149 tests for heterozygote deficiency, only the population KCHB for the locus SFb5 showed heterozygote deficiency (P<0.05), after controlling both the FWER and the FDR. Heterozygosity excess was never detected in any population and any locus. Further, the LD test showed no statistical significance for all the 666 tests between pairs of loci indicating an independent segregation of loci. The locus SFb5 for the population KCHB showed a relatively high percentage of null alleles (21%). However, the percentage of null alleles for SFb5 on average across the 15 local populations was only 4.5%. In total, null allele frequency estimates ranged from 0.6% to 4.5% with 2.5% on average across loci. The estimation of *F_ST_* both using and without using the *ENA* correction method gave equal results; *F_ST_* ^not using *ENA*^  =  *F_ST_* ^using *ENA*^  =  0.0524 with the respective 95% CI [0.039–0.072] and [0.038–0.073]. Classical measures of population differentiation are only slightly biased with a null allele frequency ranging between 5% and 8% on average across loci [50], [93]. Given that the average percentage of null allele frequency across loci (2.5%) is lower than 5%, and that the estimation of *F_ST_* did not vary after excluding null alleles, all loci were kept for further statistical analyses.

### Within-Population Genetic Diversity

**Figure 2:**
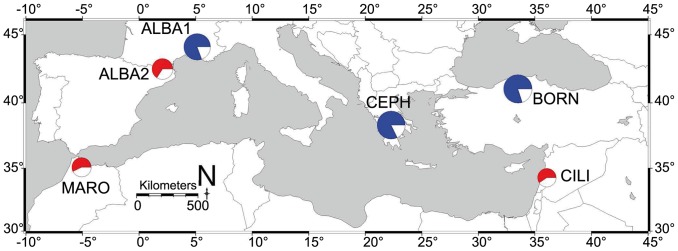
Genetic variation of *Abies cilicica* and other Mediterranean *Abies* species. Pie charts are used to depict the gene diversity corrected for sample size [55], [56] and the rarefied allelic richness [57], [58], averaged over 10 nuclear microsatellite loci, for *A. cilicica* in Lebanon (CILI), *A. bornmuelleriana* in Turkey (BORN), *A. cephalonica* in Greece (CEPH), *A. alba* in the French Southern Alps (ALBA1), *A. alba* in the French Pyrenees (ALBA2), and *A. marocana* in Morocco (MARO). Diameter of pie charts is relative to the correspondent rarefied allelic richness of the species. Proportion of red or blue color within pie charts is relative to the correspondent gene diversity of the species. Blue and red colors are employed to separate the studied fir populations into a group with significant lower genetic variation (red pie charts), and a group with significant higher genetic variation (blue pie charts) based on 10000 permutations of populations among groups performed in fstat version 2.9.3.2 [54].

In total, 109 different alleles were scored in the 351 genotyped individuals. Mean rarefied allelic richness per locus and per population averaged 4.14 (±0.02 SE) for a standardized sample size of n = 20 gene copies and 2.77 (±0.01 SE) alleles per locus and per population for a standardized sample size of n = 6 gene copies (Table 2). Mean rarefied private allelic richness per locus and per population averaged 0.15 (±0.005 SE) (Table 2). The mean observed heterozygosity (*H_O_* = 0.578±0.017 SE) was not significantly different from the mean expected heterozygosity under HWE (*H_E_* = 0.595±0.016 SE) (P>0.05) (Table 2). The overall mean inbreeding coefficient (*F_IS_* = 0.029) was not significantly different from zero with the 99% CI [−0.024–0.090] (Table 2). Permutation tests showed that *A*. *cilicica* was significantly less genetically diverse (P<0.001) and had significantly less allelic richness (P<0.001) than the studied northern Mediterranean fir species (*A. alba*, *A. bornmuelleriana* and *A. cephalonica*) (Figure 2, Table 2). Despite being significantly less genetically diverse (P<0.01) and significantly less rich in alleles (P<0.01) than *A. alba* populations from the French Pyrenees and the French Southern Alps, no significant difference was observed for gene diversity and rarefied allelic richness between populations of *A. alba* from the Pyrenees and *A. cilicica* from Lebanon (P>0.05) (Figure 2, Table 2). Similarly, *A. cilicica* and *A. marocana* were not significantly different in both gene diversity and rarefied allelic richness (P>0.05) (Figure 2, Table 2).

**Table 2:** Genetic variation and genetic distinctiveness of *Abies cilicica* and other Mediterranean *Abies* species.

Code	*H_O_*	*H_E_*	*A_R (20)_*	*A_R (6)_*	***П**_s_*	*F_IS_*
KCHB	0.568	0.616	4.95	3.04	0.18	0.079
BTJF	0.582	0.607	4.40	2.9	0.24	0.042
CHIR	0.629	0.631	4.57	2.95	0.05	0.005
JBMD	0.615	0.629	4.57	2.93	0.14	0.023
MKTF	0.561	0.612	4.32	2.85	0.22	0.086
NBSH	0.567	0.572	4.02	2.67	0.10	0.010
QARS	0.448	0.497	3.23	2.34	0.24	0.103
QJRN	0.609	0.598	4.39	2.83	0.35	−0.019
QEMN	0.603	0.585	4.33	2.78	0.18	−0.031
HNKT	0.665	0.611	4.09	2.77	0.08	−0.094
HNKF	0.621	0.619	4.47	2.91	0.20	−0.004
GHAB	0.579	0.625	4.45	2.90	0.22	0.075
HKYT	0.558	0.600	3.21	2.56	0	0.072
KMHR	0.621	0.599	3.45	2.67	0	−0.037
EHDN	0.446	0.522	3.67	2.53	0.01	0.149
Mean	0.578	0.595	4.14	2.77	0.15	0.029
MARO	0.405	0.572	-	2.86	-	-
BORN	0.668	0.799	-	4.22	-	-
CEPH	0.713	0.821	-	4.17	-	-
ALBA1	0.663	0.808	-	3.93	-	-
ALBA2	0.640	0.662	-	3.08	-	-

*H_O_* observed heterozygosity per population averaged over loci; *H_E_* expected heterozygosity corrected for sample size averaged over loci [55], [56]; *A_R (20)_* rarefied allelic richness for a sample size of 20 gene copies averaged over loci [57], [58]; *A_R (6)_* rarefied allelic richness for a sample size of 6 gene copies averaged over loci [57], [58]; *П_s_* rarefied private allelic richness for a sample size of 20 gene copies averaged over loci [57], [60]; *F_IS_* inbreeding coefficient [59]. MARO refers to *Abies marocana* in Morocco, BORN to *Abies bornmuelleriana* in Turkey, CEPH to *Abies cephalonica* in Greece, ALBA1 to *Abies alba* in the French Southern Alps and ALBA2 to *Abies alba* in the French Pyrenees. Other codes refer to *Abies cilicica* in Lebanon (See Tables 1 and S1).

### Among-Population Genetic Differentiation

The among-population genetic differentiation based on allele identities (*F_ST_*) and on allele sizes (*R_ST_*) averaged 0.0536 (P = 0) and 0.0374 (P = 0), respectively, across all loci. Allele size permutation tests showed that the observed value of *R_ST_* computed before randomization was not significantly different from the *R_ST_* obtained after allele size permutation (*pR_ST_*) (P = 0.43). Therefore, strict stepwise mutations did not contribute to among-population differentiation and no phylogeographic pattern was detected.

The Bayesian analysis of population structure in structure and calculation of Δ*K* from the structure output produced a modal value near 100 of the statistic at *K* = 2, thus indicating that the uppermost hierarchical level detected by structure was two distinct genetic clusters. The Bayesian analysis of population structure in baps split the 15 local populations into two genetically distinct groups with a posterior probability of 0.95: 11 populations (KCHB, BTJF, CHIR, JBMD, MKTF, NBSH, QJRN, QEMN, HNKT, HNKF and GHAB) were grouped into a single deme (deme 1) while the other 4 populations (QARS, HKYT, KMHR and EHDN) formed another deme (deme 2). Because of their geographic distribution, throughout this paper, the demes 1 and 2 are referred to as “Northeastern ridge” and “Southwestern ridge” respectively.

The permutation test of populations performed in fstat revealed significant differences among demes. Notably, the Southwestern ridge had significantly lower allelic richness (P<0.001), had significantly lower gene diversity (P<0.01), but significantly higher differentiation (P<0.05) than the Northeastern ridge (Table 3). The total genetic diversity of each deme, however, was not significantly different and neither was *F_IS_* (P>0.05) (Table 3). Additionally, *R_ST_* was not significantly different from *pR_ST_* for each deme (P>0.05), thus, no phylogeographic pattern was detected within the Northeastern ridge and within the Southwestern ridge (Table 3).

**Table 3:** Genetic variation, population structure and phylogeography in the Northeastern and Southwestern ridges.

	*H_O_*	*H_E_*	*A_R_*	*H_T_*	*F_IS_*	*F_ST_*	*R_ST_*	*pR_ST_*
Northeastern ridge	0.595 **	0.609**	4.413***	0.631NS	0.023NS	0.038*	0.018NS	0.024NS
Southwestern ridge	0.521 **	0.556**	3.389***	0.594NS	0.064NS	0.081*	0.0731NS	0.0858NS

*H_O_* observed heterozygosity per deme [76]; *H_E_* expected heterozygosity per deme and corrected for sample size [76]; *A_R_* rarefied allelic richness per deme for a sample size of 102 gene copies [57], [58]; *H_T_* overall gene diversity per deme [76]; *F_IS_* inbreeding coefficient per deme [59]; *F_ST_* genetic differentiation per deme based on allele identity [59]; *R_ST_* genetic differentiation per deme based on allele size [63], [64]; *pR_ST_* permuted *R_ST_* after allele size permutation test [65]. Columns 1 to 6 indicate the statistical differences in genetic variation and population structure between populations of the Northeastern ridge and populations of the Southwestern ridge. Columns 7 and 8 indicate the results of the test of phylogeographic patterns corresponding to the statistical differences between *R_ST_* and *pR_ST_* within the Northeastern ridge and within the Southwestern ridge. * P<0.05; ** P<0.01; *** P<0.001. ^NS^Non-Significant test.

**Figure 3:**
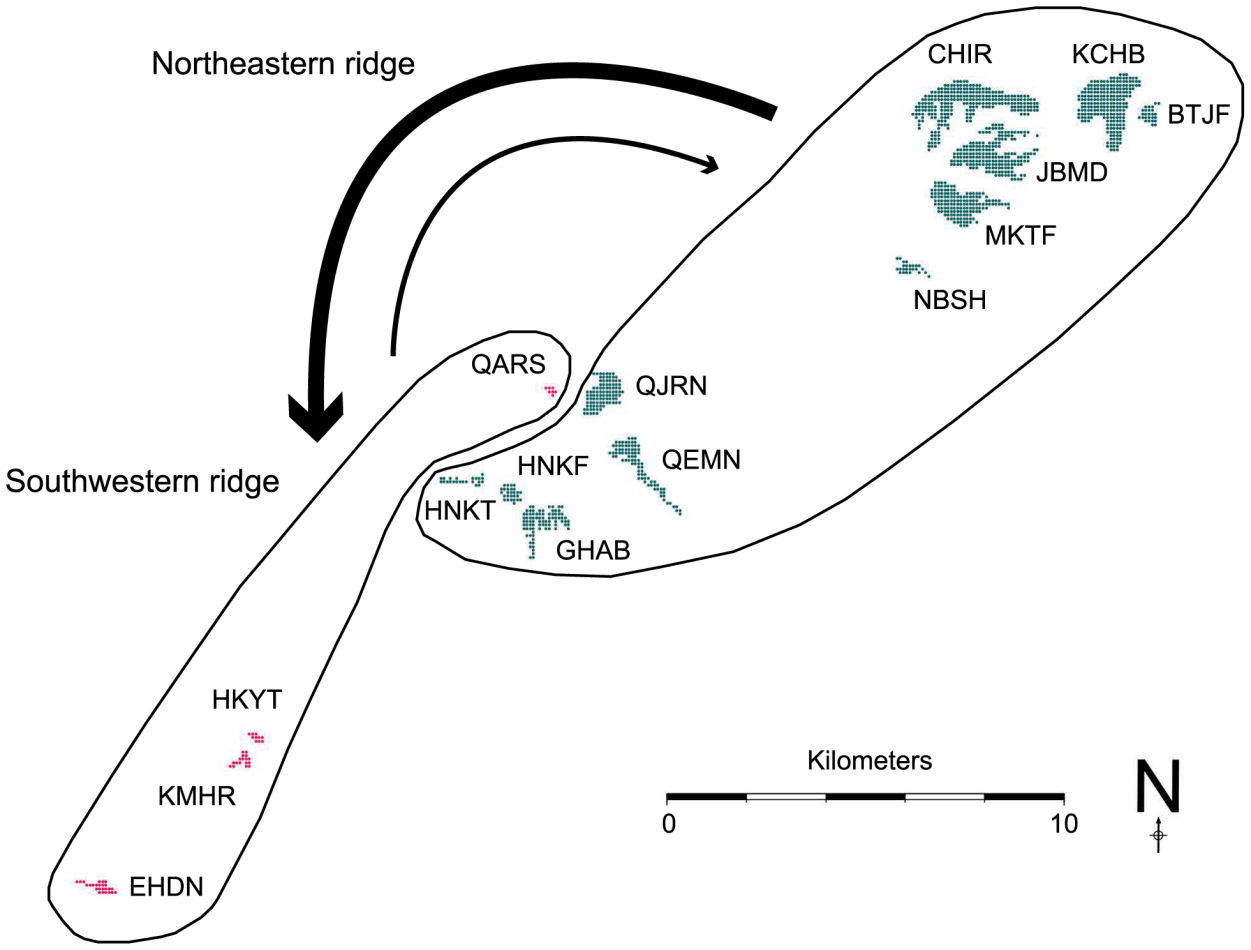
Asymmetric Northeast-Southwest migration of *Abies cilicica* in Lebanon. The Northeastern and Southwestern ridges correspond to the two genetically-distinct demes assigned by baps [71]. Populations of the Northeastern ridge are plotted in green color while those of the Southwestern ridge are plotted in magenta color. A likelihood-based Bayesian assignment test [79] performed in geneclass2 [77] allowed the detection, in the Southwestern ridge, of 3 F_0_ migrants originating from the Northeastern ridge. It has also allowed the detection, in the Northeastern ridge, of 1 F_0_ migrant originating from the Southwestern ridge, suggesting an asymmetric Northeast-Southwest migration.

The likelihood-based Bayesian assignment test performed within geneclass2 allowed the detection of 11 first-generation migrants among the 15 remnant populations of *A. cilicica* in Lebanon (P<0.01) (Table 4). Gene flow within the Northeastern ridge was pronounced with a total of 6 F_0_ migrants, although only one F_0_ migrant was detected within the Southwestern ridge. Among-deme gene flow was asymmetrical: more gene flow from the Northeastern ridge towards the Southwestern ridge (3 F_0_ migrants) and less gene flow from the Southwestern ridge towards the Northeastern ridge (1 F_0_ migrant) (Figure 3). Dispersal occurred not only between neighboring populations, but also between distant populations at distance intervals comprised between 10 Km and 20 Km. Dispersal was not observed between the two most distant local populations, i.e. KCHB and EHDN.

**Table 4:** First-generation migrants among populations and among demes.

F_0_ migrant	Sampled population	Population of origin	P-value
1	CHIR^1^	HNKF^1^	0.0084
2	MKTF^1^	HNKF^1^	0.0003
3	NBSH^1^	HNKT^1^	0.0098
4	KCHB^1^	BTJF^1^	0.0045
5	BTJF^1^	KCHB^1^	0.0049
6	QEMN^1^	GHAB^1^	0.0021
7	KMHR^2^	HKYT^2^	0.0003
8	QARS^2^	BTJF^1^	0.0000
9	EHDN^2^	QEMN^1^	0.0010
10	EHDN^2^	GHAB^1^	0.0091
11	QEMN^1^	EHDN^2^	0.0025

This table corresponds to the results of the likelihood-based Bayesian assignment test [79] performed within geneclass2 [77] allowing the detection of first-generation (F_0_) migrants within and between the Northeastern and Southwestern ridges. The probability computation at the 1% significance level was performed using the Monte Carlo resampling method of [78] with 10000 simulated individuals. Rows 1 to 6 correspond to recent dispersal between populations of the Northeastern ridge (6 F_0_ migrants) while row 7 corresponds to recent dispersal between populations of the Southwestern ridge (1 F_0_ migrant). Recent dispersal between both the Northeastern and Southwestern ridges is represented in rows 8 to 11 (4 F_0_ migrants). See Tables 1 and S1 for codes.

1 membership of the local population to deme 1 (Northeastern ridge).

2 membership of the local population to deme 2 (Southwestern ridge).

### Isolation-By-Distance

**Figure 4:**
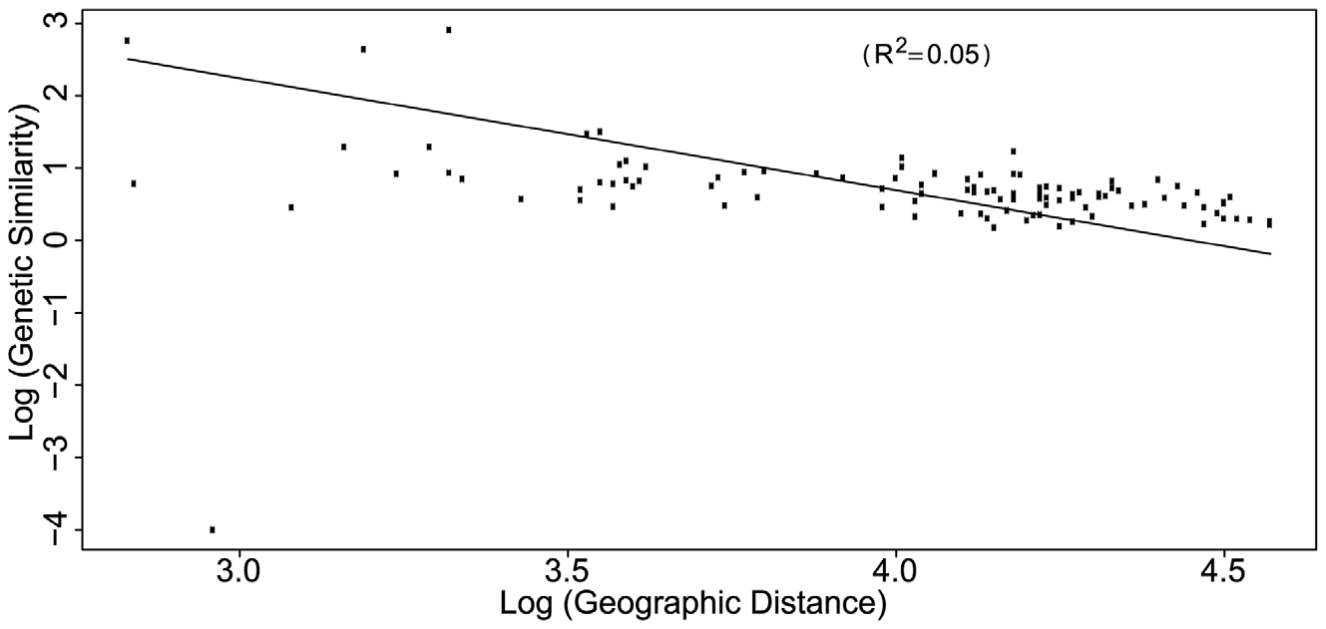
Isolation-by-distance and non-equilibrium conditions between dispersal and genetic drift. The logarithm of genetic similarity is plotted against the logarithm of geographic distance among population pairs [26]. Estimations of slope, intercept and R^2^ of the relationships were calculated using Reduced Major Axis (RMA) regression [83]. The RMA regression explained only 5% of the variance in the whole distribution area of *Abies cilicica* in Lebanon. Analysis was performed in ibdws [81].

Isolation-by-distance (IBD) assessed over all populations was significant (Mantel test, P<0.01). However, the strength of the IBD was weak, with the RMA explaining only 5% of the variance in the whole distribution area (r = −0.2248; R^2^ = 0.05) (Figure 4), thus implying non-equilibrium conditions between dispersal and genetic drift. The weak strength of IBD and the lack of phylogeographic patterns further implied a recent isolation of local populations. No pattern of IBD was detected within the Northeastern ridge (P = 0.1568) and within the Southwestern ridge (P = 0.0834).

### Genetic Bottlenecks

**Figure 5:**
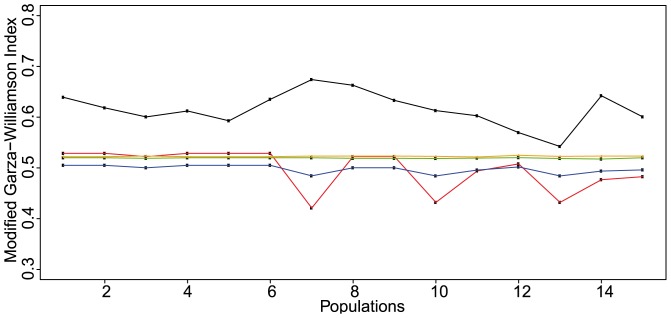
Observed and simulated values of the modified Garza-Williamson index for *Abies cilicica* populations. This figure shows the different observed values of the *M*-ratios calculated for the microsatellite loci and the values of the simulated critical *M* threshold (*M*_c_) below which a bottleneck is evident. The observed *M*-ratio values are displayed in black solid line. The *M*_c_ values calculated for *θ* = 10, *θ* = 1, *θ* = 0.5, and *θ* = 0.1 are respectively displayed in red, blue, green and orange solid lines. All the observed *M*-ratio values were significantly higher than the simulated *M*_c_ values for different pre-bottleneck *θ* values. Values of the *M*-ratio and the *M*_c_ parameters were obtained using m-p-val and critical_m [84].

**Figure 6:**
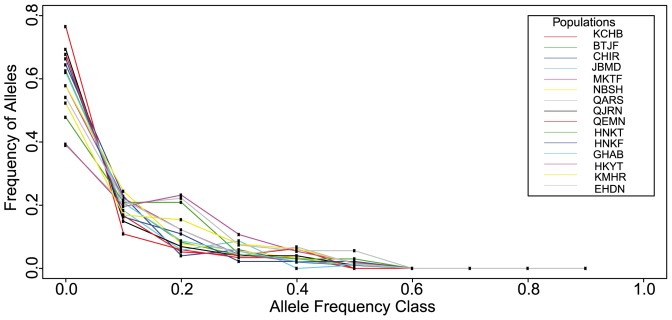
Allele frequency distribution over different allele frequency classes for each population of *Abies cilicica*. This figure shows the distribution of allele frequencies across the 15 local populations. The values were obtained using the mode-shift test [89]. The distribution of allele frequencies across the 15 local populations is plotted using different colors. All populations showed a higher abundance of rare alleles (frequency<0.1) than alleles at intermediate frequencies (0.1–0.2).

The observed values of the Garza-Williamson indices ranged between 0.54 and 0.67. However, there was no statistical evidence of a recent and severe genetic bottleneck. First, the observed *M*-ratios were not significantly lower than the simulated equilibrium distribution of *M* for all the different pre-bottleneck *θ* values (P>0.05). Second, the bottleneck threshold values, calculated for all the pre-bottleneck *θ* values, were significantly lower than the observed *M*-ratios (P<0.05) (Figure 5). The Wilcoxon's signed rank test revealed recent heterozygosity excess in only 4 populations (MKTF, P<0.05; QARS, P<0.01; HNKT, P<0.001; and KMHR, P<0.05). However, for each local population, the mode-shift test detected the typical L-shaped allele frequency distribution of non-bottlenecked populations at mutation-drift equilibrium (Figure 6). We concluded, for the 3 different tests, that no recent and severe genetic bottleneck was evident for any local population.

## Discussion

*A. cilicica* in Lebanon revealed low within-population genetic diversity (0.595±0.016 SE), low allelic richness (2.77±0.01 SE), compared to other *Abies* species, and low among-population genetic differentiation (*F_ST_* = 0.0536; *R_ST_* = 0.0374). Populations were not inbred and showed no signal of a recent and severe genetic bottleneck. Despite the low spatial genetic structure inferred from classical genetic differentiation parameters, populations showed a significant genetic divergence, forming two demes, the Northeastern and Southwestern ridges. Additionally, despite their isolation-by-distance, populations were capable of long-distance dispersal. We discuss in the following sections how these contradictory features may have emerged and their effect on the persistence of *A. cilicica* in Lebanon. We finally discuss the implications of these results for conservation.

### A Lower Within-Population Genetic Diversity than Expected

Previous comparative studies between *Abies* species in the Mediterranean, using allozymes and chloroplast microsatellites [2], [3], have respectively shown that *A. cilicica* in Turkey and Syria harbor high values of heterozygosity. High levels of genetic variation have also been found for *A. cephalonica* and *A. bornmuelleriana* in numerous studies [2]–[5], and were further supported in our study. Contrary to its geographical position in the Eastern Mediterranean and its life-history traits as a conifer species, our results have shown that *A. cilicica* in Lebanon revealed unexpected low within-population genetic diversity and low allelic richness. Particularly, this low genetic variation was not significantly different from the genetic variation of *A. marocana* in the Moroccan Rif and *A. alba* in the French Pyrenees. Previous studies have also reported low genetic variation in *A. marocana* [2], [3]. The significant lower genetic variation found in *A. alba* in the French Pyrenees with respect to *A. alba* in the French Southern Alps is also consistent with a previous study [6]. Particularly, *A. alba* in the Pyrenees is isolated within a glacial refugium and highly differentiated from the rest of its range [7]. The low genetic variation detected in *A. alba* in the Pyrenees might be the result of their exposure to a historic severe genetic bottleneck [8].

The low genetic variation of *A. cilicica* in Lebanon might be explained by the historical timber use during the Ancient Egyptian New Kingdom's rule over Phoenicia [36] and more recently during World War II [37], and by current severe illegal logging and overgrazing activities, resulting in strong habitat fragmentation. In this context, the absence of signatures of recent and severe genetic bottlenecks is unexpected and so is the high recent effective population size of *A. cilicica* in Lebanon. The low genetic variation might have been shaped over time by a rather long-term than a short-term low effective population size. One probable cause of this might be the temporal fluctuation in population sizes [22], [23], which can be typical of populations situated at the margins of geographic distributions [94]. It might also be the result of temporal variation in mating system dynamics, which can be typical of conifer species with dynamic mating systems [95], [96]. It might as well be the result of the founder effect mediated by local extinction and recolonizations in a metapopulation context [24]. Nevertheless, the genetic imprint of recent founder events within these populations might have been rapidly masked by gene flow [28], and particularly by long-distance dispersal.

### A Significant Genetic Structure despite Low *F_ST_*

Despite the low observed value of *F_ST_*, the Bayesian clustering methods detected two genetically distinct, but highly admixed demes, the Northeastern and Southwestern ridges. Populations of the Southwestern ridge inhabited the smallest habitat patches and occupied the southernmost part of the distribution area. Populations of the Southwestern ridge were significantly less genetically diverse, had significantly less allelic richness, but were significantly more genetically differentiated than populations of the Northeastern ridge. Despite these differences, the Northeastern and Southwestern ridges shared similar ecological attributes. Both demes occupied a similar elevation range, between ca. 1000 and 1800 m.a.s.l. Neither deme showed inbreeding or sub-population structure. Both demes preserved the same amount of total genetic diversity (*H_T_*), and were capable of short-distance and long-distance dispersal. The recent large effective population size and the weak IBD pattern suggest an outperformance of migration over genetic drift. Consequently, genetic drift may not be the leading evolutionary force for population differentiation in *A. cilicica* from Lebanon. In the latter case, *F*_ST_ may not be accurate [97] and molecular measures of genetic diversity may weakly predict quantitative genetic variation [98].

### High Dispersal Ability and Persistence despite Geographic Marginality

Similarly to many tree species, the genetic structure of *A. cilicica* in Lebanon may result from rapid colonization where both long-distance and short-distance dispersal are key demographic processes [15], [99], [100]. We define the observed migration type found in *A. cilicica* from Lebanon as a recent rear-edge migration with both recent colonization balancing local extinctions and recent range expansion along altitudinal gradients. Considering that brief episodes of range expansions are thought to leave strong and persistent signature on the genetic structure of natural populations [101], the Northeast-Southwest structure of *A. cilicica* in Lebanon suggests two plausible demographic processes promoting persistence.

First, the Northeast-Southwest structure might result from recent colonization balancing local extinctions. The likelihood-based Bayesian assignment test detected an emigration rate from the Northeastern ridge exceeding three-fold that from the Southwestern ridge (Figure 3). Although, this asymmetric migration might be a simple consequence of the smaller size of the Southwestern ridge, gene flow from more genetically diverse populations may counteract drift and contribute to restoring genetic diversity in populations of the Southwestern ridge (e.g. QARS and EHDN), thus making persistence possible.

Second, the Northeast-Southwest structure might also result from recent range expansion along altitudinal gradients. Private alleles in both the Northeastern and Southwestern ridges could indicate the presence of different glacial microrefugia in both demes (Table 2). In the Northeastern ridge, more or less all local populations harbored private alleles, both at low and high elevations, suggesting multiple low-elevation and high-elevation microrefugia. The higher genetic diversity of Northeastern ridge populations might result from the mixing of these different microrefugial lineages along mountain slopes during recolonization. In the Southwestern ridge, high private allelic richness was only found in low-elevation population QARS. Other Southwestern ridge populations, located at higher elevations, harbored either no private alleles (HKYT and KMHR) or very few private alleles (EHDN), suggesting a postglacial colonization from QARS upwards. Range expansion along altitudinal gradients may have occurred concomitantly or at different time scales from the Northeastern-Southwestern asymmetric migration described earlier. Microrefugial populations may be crucial for predicting climate-change-related range shifts of tree species [102], and particularly, altitudinal range shifts may be an efficient mechanism for populations to cope with changing environmental and/or climatic conditions. A recent meta-analysis study showed that the distribution of species had recently shifted to higher elevations at an average rate of 11 meters per decade [103].

### Implications for Conservation

Because they contain most of the genetic diversity of *A. cilicica* in Lebanon, and have shaped current diversity via long-distance migration and altitudinal range shifts, all local populations of the Northeastern ridge as well as QARS of the Southwestern ridge have disproportionate conservation importance. The very low census size of QARS and its location on the lowest elevation warrant special attention. Conserving other Southwestern ridge populations (such as EHDN) would also be useful as they are stepping stones for gene flow between the two demes. EHDN is an interesting example for highlighting conservation challenges. A mixed fir/cedar population, EHDN has been managed as a nature reserve since 1992. The low number of surviving fir trees in this nature reserve as well as their low genetic variation suggest that a change of strategy is needed if conserving the genetic diversity of *A. cilicica* is a target for management. Dynamic conservation of genetic diversity [104] *in situ* requires that a particular attention should be given to regeneration and to avoid introducing seeds or seedlings from exotic locations outside demes or farther away. Attention should be given to keeping gene flow between the Northeastern and Southwestern ridges at reasonable rates. This can be potential through maintaining historic-likewise levels of gene flow by (1) preserving the natural ecological corridor between the Northeastern and Southwestern ridges, and (2) preserving a buffer zone across each local population to facilitate the natural recolonization of local populations over unoccupied habitat patches. For local populations with very low census size and genetic diversity, *ex situ* conservation (e.g. establishing seed banks and clonal archives) or assisted migration to suitable locations where future climate should be favorable to *A. cilicica* is strongly advisable.

## Conclusions

The population genetic diversity and structure of *A. cilicica* in Lebanon is at odds with biogeographical and life history trait expectations. Rather, demographic processes linked to current habitat fragmentation and geographic range structure, and to original historical range contraction and recolonization better explain genetic diversity. *A. cilicica* in Lebanon is made of two demes moderately connected by geneflow shaping a weak isolation-by-distance pattern. Persistence of *A. cilicica* in Lebanon was linked to two plausible demographic processes, occurring concurrently or sequentially, which we called rear-edge type migration: (1) alternating colonization and extinction events and (2) range shifts along altitudinal gradients. These two demographic events might help us better understand the species response to expected climate change. Populations of the Northeastern and Southwestern ridges might harbor a large evolutionary potential, and should be consequently prioritized for conservation.

## Supporting Information

Table S1 **Origin, location sample size of** ***Abies cilicica*** **and other Mediterranean** ***Abies*** **species.** Legend: Origin, location, and sample size of the 15 populations of *Abies cilicica* in Lebanon and other Mediterranean *Abies* species. Altitude is in meters above sea level ( m.a.s.l.) and the surface of the sampled populations of *Abies cilicica* in Lebanon is in hectares. N.B. Surface is not indicative of population density.
(XLS)
